# Supplementary material for: Cost-effectiveness analysis of a maternal pneumococcal vaccine in low-income, high-burden settings such as Sierra Leone
Source: PLOS Glob Public Health. 2023 Aug 24;3(8):e0000915. doi: 10.1371/journal.pgph.0000915 (PMC10449127; doi:10.1371/journal.pgph.0000915)
Supplement: S1 Text — (DOCX) [file pgph.0000915.s001.docx]

# S1. Dynamic transmission model

This section provides a summary of the dynamic transmission model underlying this paper’s cost-effectiveness analysis. Further detail on the design and execution of the model can be found in the previously published paper and Supplementary Material [1].

The dynamic transmission model uses a Susceptible-Infected-Susceptible structure, subdivided by age, immunity gained through infant PCV vaccination, and immunity gained through maternal PPV vaccination (Figure S1). The model focused on understanding the impact of a maternal pneumococcal model on pneumococcal disease in children under two, hence, the age-structure of our model included: twenty-four age classes (0, 1, 2, ..., 23 months) for children under two, one for children 2-5 years, and one for all individuals aged over 5 years.

We modelled maternally derived immunity as waning exponentially using data recorded by Holmlund and colleagues on antibody responses to multiple serotypes [2]. Based on this data, maternally derived immunity waned completely after one year. When present, the effectiveness of maternally derived immunity was assumed to be a constant ($e_{m})$ at 75%. We did not include the protective effects of a maternal vaccine for mothers, or reduction in transmission from mothers to infants. This would have required a more computationally intensive model such as an agent-based model.


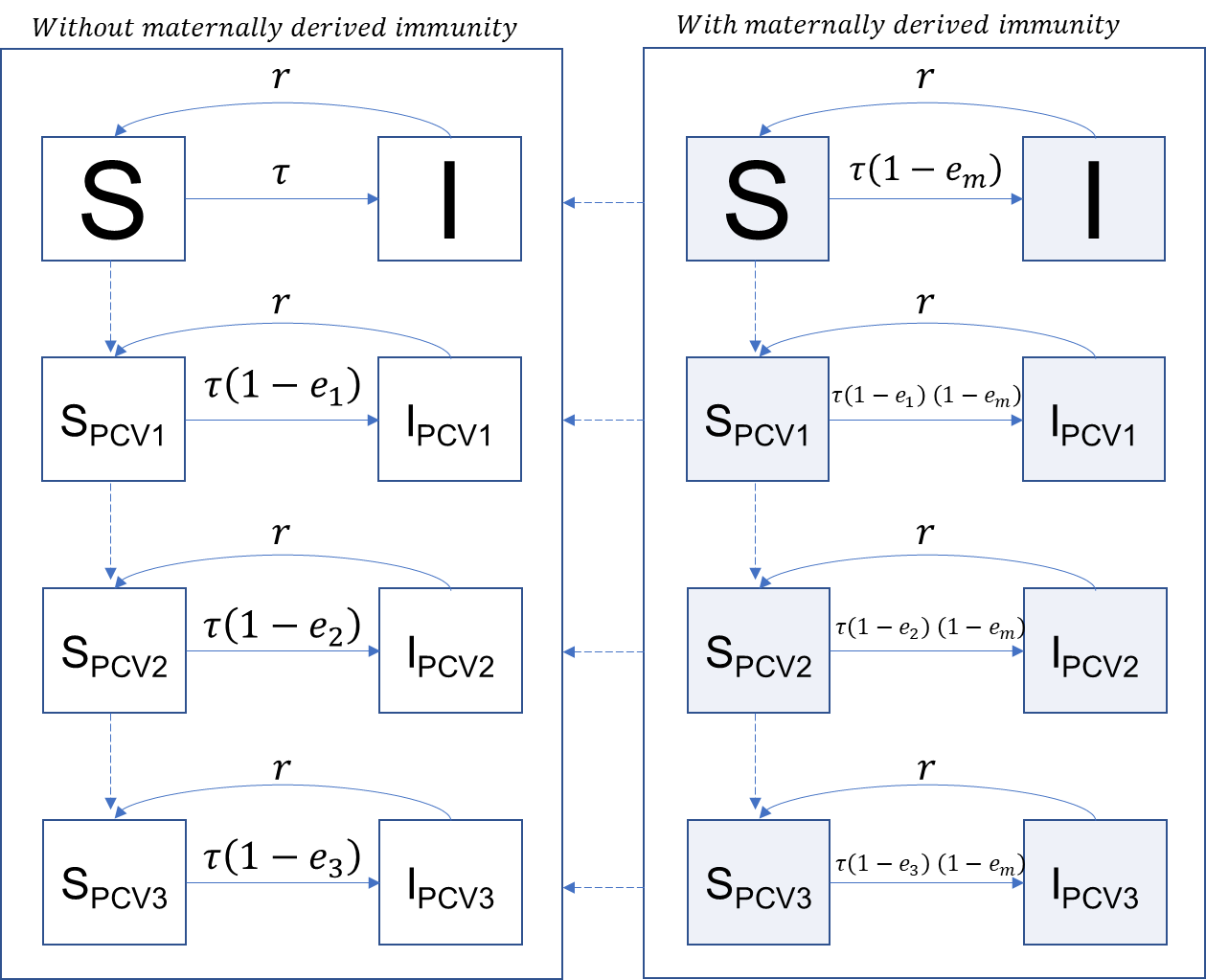


**Figure S1.** Schematic representing classes for one age group of the dynamic transmission model. Disease classes – S = Susceptible and I = Infected – include subscripts tracking immunity from doses of infant PCV. Transmission ($\tau)$ is modulated by immunity from PCV ($e_{i})$ and maternally derived immunity ($e_{m})$ for children whose mothers were vaccinated during pregnancy.

# Reference List

1. Bilgin GM, Lokuge K, Glass K. Modelling the impact of maternal pneumococcal vaccination on infant pneumococcal disease in low-income settings. Vaccine. 2022;40(31):4128-34.

2. Holmlund E, Nohynek H, Quiambao B, Ollgren J, Kayhty H. Mother-infant vaccination with pneumococcal polysaccharide vaccine: Persistence of maternal antibodies and responses of infants to vaccination. Vaccine. 2011;29(28):4565-75.
