# Supplementary figures and images for: Cost-effectiveness analysis of a maternal pneumococcal vaccine in low-income, high-burden settings such as Sierra Leone
Source: PLOS Glob Public Health. 2023 Aug 24;3(8):e0000915. doi: 10.1371/journal.pgph.0000915 (PMC10449127; doi:10.1371/journal.pgph.0000915)

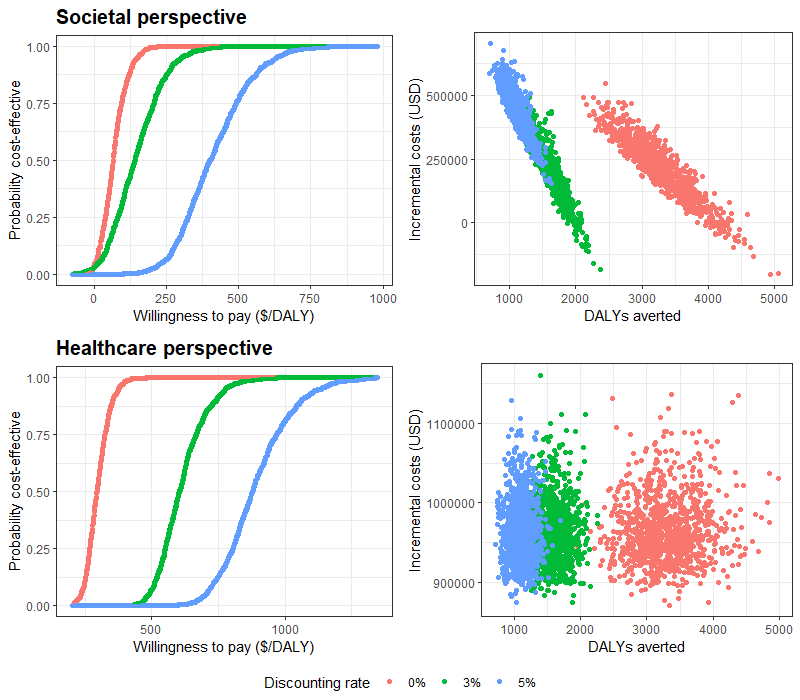

Supplement: S1 Fig — Cost-effectiveness acceptability curve (left) and expected incremental costs and disability-adjusted life years (DALYs) averted (right) for a maternal pneumococcal vaccine in Sierra Leone using varying discounting rates on costs and health outcomes. Points represent 1,000 samples drawn with probabilistic sensitivity analysis. (TIFF) [file pgph.0000915.s004.tiff]

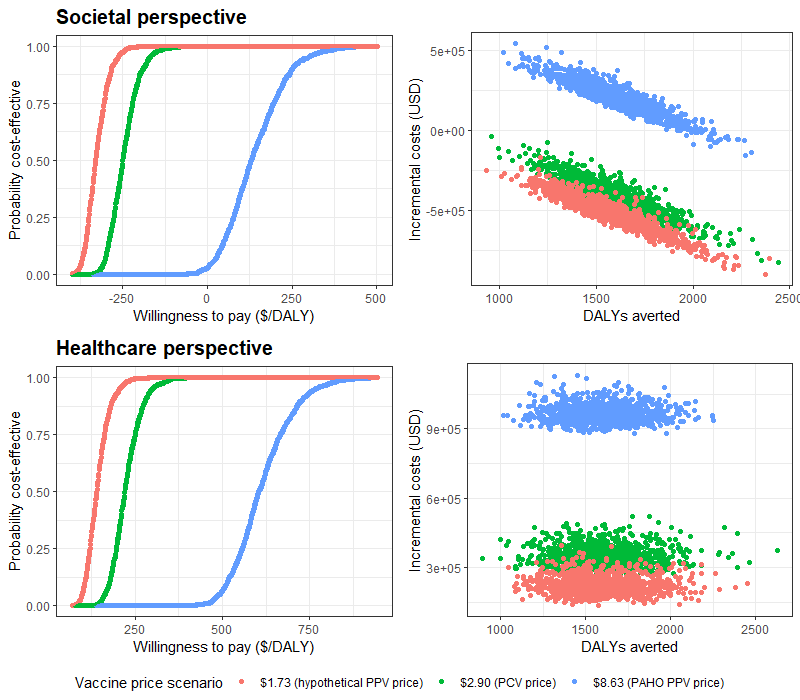

Supplement: S2 Fig — Cost-effectiveness acceptability curve (left) and expected incremental costs and disability-adjusted life years (DALYs) averted (right) for a maternal pneumococcal vaccine in Sierra Leone using varying vaccine price estimates. Points represent 1,000 samples drawn with probabilistic sensitivity analysis. (TIFF) [file pgph.0000915.s005.tiff]

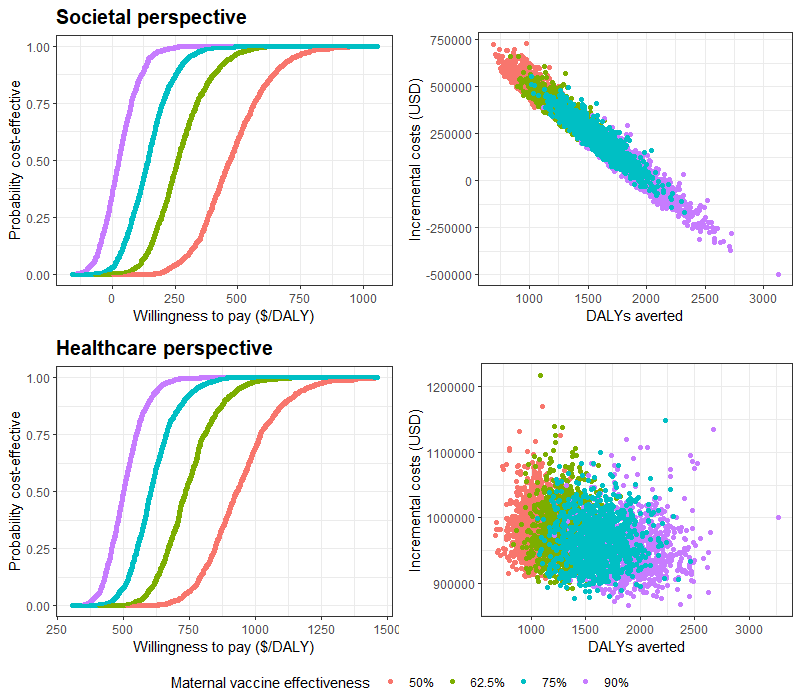

Supplement: S3 Fig — Cost-effectiveness acceptability curve (left) and expected incremental costs and disability-adjusted life years (DALYs) averted (right) for a maternal pneumococcal vaccine in Sierra Leone varying the effectiveness of the maternal vaccine in preventing severe outcomes in infants. Points represent 1,000 samples drawn with probabilistic sensitivity analysis. (TIFF) [file pgph.0000915.s006.tiff]
