# Supplementary material for: Cost-effectiveness analysis of a maternal pneumococcal vaccine in low-income, high-burden settings such as Sierra Leone
Source: PLOS Glob Public Health. 2023 Aug 24;3(8):e0000915. doi: 10.1371/journal.pgph.0000915 (PMC10449127; doi:10.1371/journal.pgph.0000915)
Supplement: Attachment — Submitted filename: 2023_05_26 Response to Reviewers.docx [file pgph.0000915.s007.docx]

We thank the reviewers for their detailed comments. We have clarified sections of our paper, included additional results, and created a Supplementary Material based on these comments. We provide line number references for the marked-up version of the manuscript in this response letter.

**Reviewer #1**

**Comment 1: The major concern is that the study was performed merely from the health system perspective. The second panel on cost-effectiveness in health and medicine has recommended that the cost-effectiveness analysis be conducted from both societal perspective and health perspective.**

We thank the reviewer for encouraging additional analysis from a societal perspective and have included analysis from a societal perspective alongside the healthcare perspective throughout the paper. The main changes to make this possible are highlighted below:

*(Excerpt from the Abstract)*

*Our model estimates that a maternal pneumococcal program would cost $606 (2020 USD, 95% prediction interval 437 to 779) from a healthcare perspective and $132 (95% prediction interval -1 to 265) from a societal perspective per DALY averted) for one year of vaccine delivery. Hence, a maternal pneumococcal vaccine would be cost-effective from a societal perspective but not cost-effective from a healthcare perspective using Sierra Leone’s GDP per capita of $527 as a cost-effectiveness threshold. [Line 27]*

*(Excerpts from the Methods)*

*We assumed that maternal pneumococcal vaccination would take place as part of routine antenatal care visits and therefore did not include direct non-medical costs such as patient travel time. [Line 150]*

*We estimated direct and indirect medical costs using a Ghanaian costing study of pneumococcal pneumonia and meningitis. [Line 206]*

*We considered the loss of lifetime productivity due to premature death, and reduced productivity of caregivers while caring for infants using a human capital approach (Table 2) [1]. Lost productivity due to premature death was estimated using UN estimates of life expectancy and World Bank estimates of gross national income per capita. Estimates for lost productivity of caregivers was taken from the Ghanaian study. We did not include productivity losses due to long-term sequalae. [Line 217]*

*[Additional rows in Table 2]*

| *Parameter* | *Presentation* | *Base estimate* | *Distribution* | *Reference* |
| --- | --- | --- | --- | --- |
| *Direct non-medical costs* | *Pneumococcal meningitis inpatient* | *$1.95* | *Uniform(0.41,2.98)** | *[2]* |
|  | *Pneumococcal pneumonia or NPNM inpatient* | *$0.62* | *Uniform(0.00,1.54)** | *[2]* |
|  | *Outpatient* | *$0.00* | *Uniform(0.00,0.41)** | *[2]* |
| *Caregiver productivity loss* | *Pneumococcal meningitis* | *$36.28* | *Uniform(11.7,61.1)** | *[2]* |
|  | *Pneumococcal pneumonia* | *$13.05* | *Uniform(3.29,48.8)** | *[2]* |
| *Productivity loss due to premature death* | *Death* | *$13,282 (3% discounting)*  *$27,401 (0% discounting)*  *$9,202 (5% discounting)* | *NA* | *[3, 4]* |

*(Excerpts from the Results)*

*A maternal pneumococcal vaccine appears cost-effective from a societal perspective ($132 per DALY averted) but not from a healthcare perspective ($606 per DALY averted) using Sierra Leone’s GDP ($527) as a threshold for cost-effectiveness (Table 3). [Line 250]*

*The cost of delivering the vaccine program ($1,022,305 per 100,000 infants) had a greater influence compared to health system costs averted ($69,376 per 100,000 infants) over the expected incremental cost-effectiveness of the vaccine from a healthcare perspective… Productivity loss due to premature mortality accounted for a majority ($881,532, 92.6%) of costs saved by a maternal pneumococcal vaccine from a societal perspective (Table S2). [Line 258]*

*Sensitivity analysis identified health outcome discounting rates, vaccine price and vaccine effectiveness to be highly influential over cost per DALY averted (Fig 3 and Fig S2-4 in the Supplementary Material). Without discounting, the expected cost per DALY averted was cost-effective from both a healthcare perspective ($292, 55% of Sierra Leone’s GDP) and a societal perspective ($66, 13% of Sierra Leone’s GDP per capita). The vaccine would be cost-effective from a healthcare perspective ($142, 27% of Sierra Leone’s GDP) and cost saving from a societal perspective if PPV could be procured at the same cost relative to PCV in Sierra Leone as on the PAHO reference price list. The vaccine would be cost-effective from a healthcare perspective under current market prices with an effectiveness of at least 85%. [Line 279]*

*(Excerpts from the Discussion)*

*With current market prices, a maternal pneumococcal vaccine would be cost-effective from a societal perspective but not be cost-effective from a healthcare perspective in Sierra Leone using Sierra Leone’s GDP per capita of $527 (2020 USD) as a cost-effectiveness threshold [5]. [Line 296]*

*Productivity losses due to premature mortality were the main influence determining the difference between cost-effectiveness estimates from a societal and healthcare perspective, aligning with previous analysis of pneumococcal interventions in Ghana [2]. [Line 304]*

**Comment 2: Another major concern is the reporting does not confirm CHEER good practice. Please check it. Some key parameters are missing, which does not allow other others to repeat the analysis. For example,**

**a. How will the vaccine be vaccinated among pregnant women?**

**b. What are efficacy rates applied to different age of the children?**

**c. What are the incidence rates of pneumococcal-associated illness among children under 1 month, and children between 1-2 months,**

**d. What is the share of conditions under different treatment settings and their associated health outcomes (e.g., death rate)**

We thank the reviewer for directing us to the CHEER good practice guidelines. We have read the guidelines and included additional detail throughout the paper to ensure that we adhere to them.

*(Excerpt from the Introduction)*

*Addressing reviewer query (a): We consider a year-round programme for maternal pneumococcal vaccination which, similar to maternal tetanus toxoid vaccination in low-resource settings, opportunistically vaccinates pregnant women who present to antenatal care. [Line 69]*

*(Excerpts from the Methods)*

*CHEER guideline #7 - explicitly stating the comparator: In this study, we quantify the incremental cost-effectiveness of introducing a maternal PPV vaccine alongside an infant PCV schedule compared to continuing with infant PCV vaccination only. [Line 76]*

*CHEER guideline #9 - explicitly stating the time horizon of the study and why it is appropriate: The time horizon of the study was one year since maternally derived immunity is expected to wane after one year [6], and a maternal pneumococcal vaccine is anticipated to only affect the incidence of disease in infants, not cause wider or ongoing reductions in transmission [7]. [Line 84]*

*CHEER guideline #10 - explicitly reporting choice of discounting rate: We applied a discounting rate of 3% was applied to all health benefits and costs in line with existing cost-effectiveness analysis in Sub-Saharan Africa [8-11]. Discounting was applied using a continuous time approach with uniform age weighting [12]. [Line 87]*

*CHEER guideline #20 - explicitly stating how we visualised uncertainty: Probabilistic sensitivity analysis was conducted by Monte Carlo simulation, that is, by running the model 1000 times randomly drawing per-patient from the probability distributions of all underlying health outcome and costing parameters. The corresponding 1000 cost and 1000 health effect values were presented on a cost-effectiveness plane and summarised by a cost-effectiveness acceptability curve. [Line 240]*

*(Additional sections)*

*CHEER guideline #16 - reporting where the model is publicly available: We collated all data from publicly available data sources. All data and code can be viewed on our GitHub:* [*https://github.com/gizembilgin/SLE_maternalPneumococcal_model*](https://github.com/gizembilgin/SLE_maternalPneumococcal_model) *[Line 372]*

*CHEER guideline #27 - making the influence of funding sources explicit: GMB was supported by the Australian Government Research Training Program during this study. This scholarship did not influence the design of the study, writing of the manuscript, or decision to submit for publication. [Line 376]*

*(Excerpts from the Supplementary Material)*

*Addressing reviewer query (b): We modelled maternally derived immunity as waning exponentially … [over] one year. When present, the effectiveness of maternally derived immunity was assumed to be constant at 75%. [page 2]*

*In relation to the reviewer’s queries (c) and (d):*

(c) We have created a script – answer to Reviewer 1’s query – in our publicly available GitHub which provides the incidence rates of pneumococcal-associated illness for each of the model’s twenty-six age groups and five severe outcomes of pneumococcal disease. The incidence rates of pneumococcal-associated illness among children under 1 month, and children between 1-2 months are:

(d) Unfortunately, there is insufficient data in Sierra Leone on the death rates (for example) under different treatment settings. We reviewed available literature and could only find studies in Sierra Leone for health outcomes by treatment settings for Ebola, likely due to the funding associated with the Ebola outbreak response. Hence, we did not model deaths by treatment setting and assumed a constant cost by treatment setting regardless of whether the patient was discharged or passed away in care.

**Comment 3: Regarding the cost, health care cost is higher than outpatient cost, which seems not reasonable. Also, the cost of outpatient costs less than 1 dollar is not reasonable either. Please note that WHO-CHOICE data do not include the cost of medicine and lab tests. It is not clear whether those costs are converted to 2020 USD.**

We have updated our estimates of outpatient costs from WHO-CHOICE data to estimates from a Ghanian costing study which includes a broader range of medical costs such as the cost of medicine and lab tests. This has increased our estimate of outpatient costs from $0.95 to $1.03.

We are unsure whether the reviewer is referring to inpatient costs in this comment when they suggest that healthcare costs being higher than outpatient costs are unreasonable. We expect the costs of patients admitted to hospital (inpatients) to be higher than patients who are treated without admission (outpatients).

|  | Base estimate | Distribution | Reference |
| --- | --- | --- | --- |
| Previous estimate for an outpatient visit | $0.95 | Gamma(1.6,0.59) | [13, 14] |
| Updated estimate for an outpatient visit | $1.03 | Uniform(0.00,2.98)* | [2] |

* Distribution as per correspondence with Ghanaian study author [2]

**Comment 4: It would be better to report outcomes under two scenarios (no vaccine and with vaccine), which could help readers understand better whether the estimation is reasonable, instead of ICER directly.**

We have included a table in the Supplementary Material which provides this detail.

*(Excerpt from the Supplementary Material – page 4)*

***Table S2.*** *Breakdown of expected costs and health outcomes with and without a maternal pneumococcal vaccine. Outcomes presented for as the mean result over 1,000 simulations for 100,000 children.*

|  | ***Without maternal vaccine*** | ***With maternal vaccine*** | ***Difference*** |
| --- | --- | --- | --- |
| ***Incidence of health outcomes*** |  |  |  |
| *Cases* | *102,985* | *91,613* | *11,372* |
| *Invasive pneumococcal disease* | *1,935* | *1,549* | *386* |
| *Pneumococcal meningitis* | *78* | *57* | *21* |
| *Pneumococcal pneumonia* | *1,650* | *1,325* | *325* |
| *Pneumococcal-attributable hospitalisation* | *1,296* | *1,037* | *259* |
| *Death* | *243* | *187* | *56* |
| ***Direct medical costs*** |  |  |  |
| *Pneumococcal meningitis* | *13,435* | *9,753* | *3,682* |
| *Pneumococcal pneumonia* | *270,711* | *217,489* | *53,222* |
| *Outpatient* | *4,622* | *3,406* | *1,216* |
| *Home care* | *742* | *547* | *195* |
| ***Direct non-medical costs*** |  |  |  |
| *Pneumococcal meningitis* | *101* | *74* | *27* |
| *Pneumococcal pneumonia* | *952* | *766* | *186* |
| *Outpatient* | *638* | *470* | *168* |
| *Home care* | *0* | *0* | *0* |
| ***Productivity loss*** |  |  |  |
| *Caregiver of infant with pneumococcal meningitis* | *2,181* | *1,580* | *601* |
| *Caregiver of infant with pneumococcal pneumonia* | *32,174* | *25,880* | *6,294* |
| *Premature mortality* | *3,224,048* | *2,479,043* | *745,005* |
| ***Maternal vaccine costs*** | *0* | *953,717* | *-953,717* |
| ***Net cost (healthcare perspective)*** | *289,510* | *1,184,913* | *-895,403* |
| ***Net cost (societal perspective)*** | *3,549,604* | *3,692,725* | *-143,121* |

**Comment 5: The 95% CI of ICER is quite narrow. Given there are so many parameters included in the analysis, the accuracy of the estimation is skeptical.**

We have checked how the uncertainty of each parameter is propagated through our model.

First, we considered all parameters listed in this cost-effectiveness paper. Previously we had sampled per individual from the distribution of all parameters. We have now moved from individual-based sampling of operational costs to a program-based sampling of operational costs. The meta-analysis informing this parameter estimated the distribution of operation costs across vaccination programs, not the distribution of operational costs across individuals within vaccination programs. Sampling once, rather than per individual, across each model run has widened the prediction intervals around our model results.

Second, we considered how outputs from the underlying transmission model passed through to the cost-effectiveness model. We had not previously included uncertainty in the severity of pneumococcal outcomes in children in our cost-effectiveness estimates. We have now included this uncertainty in cost-effectiveness estimates.

The effect of these two amendments have been propagated throughout the updated results, discussion, and abstract of the paper. Our model now captures all possible sources of uncertainty in parameters informing cost-effectiveness estimates.

**Comment 6: The conclusion is worth debating even with the results of $570/DALYs. There are different cut-offs have been proposed, including one published in Lancet Global Health that uses 1.5 GDP/capita as cut-off. The choice of threshold should be discussed.**

We agree with the reviewer that the choice of cost-effectiveness threshold is worth considering. Hence, we have included a cost-effectiveness acceptability curve in the results (Figure 2) which presents the probability of a maternal vaccine being cost-effective under different cut-offs. We have also updated the text in the discussion to acknowledge that whether a maternal vaccine is considered cost-effective will depend on the decision maker’s choice of cost-effectiveness threshold, influenced by budget restrictions and local values.

*(Excerpt from the Abstract)*

*Hence, a maternal pneumococcal vaccine would be cost-effective from a societal perspective but not cost-effective from a healthcare perspective using Sierra Leone’s GDP per capita of $527 as a cost-effectiveness threshold. [Line 30]*

*(Excerpt from the Results)*

*Fig 2 presents the probability of a maternal pneumococcal vaccine being cost-effective with different cost-effectiveness thresholds and visualises uncertainty in expected incremental costs and DALYs averted. [Line 254]*

*(Excerpt from the Discussion)*

*The choice of cost-effectiveness threshold and perspective should depend on the decision-maker’s budget restrictions and local value judgements [15]. [Line 299]*

**Reviewer #2**

**Comment 1: What are the incremental costs and incremental health benefits of implementing the maternal programme? This needs to be explicitly stated front and centre. Further, a cost-effectiveness acceptability curve/frontier would be a great assessment of this study so that the readers can see how the cost-effectiveness changes given a varied wtp threshold. The standard of using GDP per capita as a fixed measure of the cost-effectiveness threshold is dated and having this information in the form of a curve with varying wtp thresholds would be much more informative for decision-makers. In addition, a plot showing the uncertainty is crucial, something like a CEA plane using ICERs or Incremental Net Monetary Benefit or a plot of the EPVI/EPPVI given a wtp threshold would greatly enrich the study. A couple of papers which the authors might find useful are (note neither of these papers is affiliated with the reviewer):**

[**https://pubmed.ncbi.nlm.nih.gov/31104743/**](https://pubmed.ncbi.nlm.nih.gov/31104743/)
[**https://journals.sagepub.com/doi/full/10.1177/0272989X211045070**](https://journals.sagepub.com/doi/full/10.1177/0272989X211045070)

We thank the reviewer for providing us with insightful references. We have updated our results to reflect recommendations by Bilcke and Beutels (2022) for reporting cost-effectiveness results with uncertainty for the comparison of two interventions. We have included a cost-effectiveness acceptability curve as requested by the reviewer (Figure 2, left). We have also included expected incremental costs and incremental health benefits as additional columns in Table 3 and visualised the uncertainty in these incremental differences by plotting the 1000 simulations on a cost-effectiveness plane (Figure 2 right).

*(Excerpt from the Results)*

***Fig 2.*** *Cost-effectiveness acceptability curve (left) and expected incremental costs and disability-adjusted life years (DALYs) averted (right) for a maternal pneumococcal vaccine in Sierra Leone. Points represent 1,000 samples drawn with probabilistic sensitivity analysis.*

***Table 3.*** *Cost per outcome averted under base model assumptions with 95% prediction interval from probabilistic sensitivity analysis. All costs presented in 2020 USD.*

| ***Outcome averted*** | ***Expected incremental cost*** | ***Expected incremental effect*** | ***Expected ICER of outcome*** | ***ICER 95% prediction interval*** |
| --- | --- | --- | --- | --- |
| ***Healthcare perspective*** | | | |  |
| *DALY* | *966,802* | *1,595* | *606* | *437 to 779* |
| *Case* | *996,802* | *948* | *1,017* | *937 to 1,096* |
| *Hospitalisation* | *966,802* | *258* | *3,740* | *2,710 to 4,779* |
| *Death* | *966,802* | *53* | *17,218* | *12,239 to 22,344* |
| ***Societal perspective*** | | | |  |
| *DALY* | *211,455* | *1,595* | *132* | *-1 to 265* |
| *Case* | *211,455* | *948* | *223* | *5 to 441* |
| *Hospitalisation* | *211,455* | *258* | *821* | *-12 to 1,660* |
| *Death* | *211,455* | *53* | *3,764* | *-46 to 7,549* |

**Comment 2: Though probably mentioned in their model paper, there needs to be some further information in this paper on how the maternal vaccine is implemented in a dynamic model for completeness. E.g. does the model take into account the protection against pneumococcus in mothers themselves? If so, how? Does the model consider the potential reduction in pneumococcal transmission between mother and infant? What are the indirect effects in total like? A compartmental figure showing this in an Appendix would be very useful. How was the waning introduced? What is the waning function in the dynamic model (this is a crucial component) it says 1 year but is this lost exponentially?**

We have created a Supplementary Material document and included a page-length summary of the dynamic transmission model containing a schematic of the model and describing the implementation of maternally derived immunity. We have ensured that the detail in this summary is sufficient to answer all of Reviewer 2’s queries in this comment.

*(Excerpt from the Supplementary Material – page 2)*

# *S1. Dynamic transmission model*

*This section provides a summary of the dynamic transmission model underlying this paper’s cost-effectiveness analysis. Further detail on the design and execution of the model can be found in the previously published paper and Supplementary Material [7].*

*The dynamic transmission model uses a Susceptible-Infected-Susceptible structure, subdivided by age, immunity gained through infant PCV vaccination, and immunity gained through maternal PPV vaccination (Figure S1). The model focused on understanding the impact of a maternal pneumococcal model on pneumococcal disease in children under two, hence, the age-structure of our model included: twenty-four age classes (0, 1, 2, ..., 23 months) for children under two, one for children 2-5 years, and one for all individuals aged over 5 years.*

*We modelled maternally derived immunity as waning exponentially using data recorded by Holmlund and colleagues on antibody responses to multiple serotypes [6]. Based on this data, maternally derived immunity waned completely after one year. When present, the effectiveness of maternally derived immunity was assumed to be a constant (*$e_{m})$ *at 75%. We did not include the protective effects of a maternal vaccine for mothers, or reduction in transmission from mothers to infants. This would have required a more computationally intensive model such as an agent-based model.*

***Figure S1.*** *Schematic representing classes for one age group of the dynamic transmission model. Disease classes – S = Susceptible and I = Infected – include subscripts tracking immunity from doses of infant PCV. Transmission (*$\tau)$ *is modulated by immunity from PCV (*$e_{i})$ *and maternally derived immunity (*$e_{m})$ *for children whose mothers were vaccinated during pregnancy.*

**Comment 3: What is the time horizon for this study? It appears to be one year? Why? Depending on how the waning of immunity is implemented, there could be multi-seasonal effects no? A much longer time horizon needs to be considered, at least 10 years, unless the authors have a good reason why(?) Further, the discounting needs to be applied to health benefits and costs. The conclusion that the choice of discounting rate is significant in determining cost-effectiveness is flawed if only implied to the DALYs, it implies that the choice of DALYs is significant in determining cost-effectiveness.**

We agree with the reviewer and have checked that discounting was applied to all ongoing health benefits and costs. We have also included an explanation of why the time horizon of the study was limited to one year.

*(Excerpt from the Methods)*

*The time horizon of the study was one year since maternally derived immunity is expected to wane after one year [6], and a maternal pneumococcal vaccine is anticipated to only affect the incidence of disease in infants, not cause wider or ongoing reductions in transmission [7]. [Line 84]*

**Comment 4: Is the code accessible in any way? Neither this paper nor the modelling paper it references has its code online to share to check for reproducibility. Modelling papers, including cost-effectiveness analysis, should have their code freely available. This work should not be in a peer-reviewed journal if the code cannot be peer-reviewed.**

We agree with the reviewer and have publicly released our GitHub repository, including version control history, available at: <https://github.com/gizembilgin/SLE_maternalPneumococcal_model>.

We believe that releasing our code repository (including input files) also addresses **PLOS requirement #3 “Have the authors made all data underlying the findings in their manuscript fully available?”** for which two reviewers responded “no”.

**Comment 5: The only programme considered appears to be a year-round maternal programme (not sure this is made explicit in the methods)? Would a seasonal programme be feasible in Sierra Leone (I’m not familiar with the epi there sorry!)? Perhaps a brief overview of the epi in the introduction would help contextualise the work within the global landscape.**

We have now made it explicit in the paper that our model considers a year-round maternal programme which opportunistically vaccinates women who present to antenatal care, like current maternal tetanus programs in low-resource settings.

*(Excerpt from the Introduction)*

*We consider a year-round programme for maternal pneumococcal vaccination which, like maternal tetanus toxoid vaccination in low-resource settings, opportunistically vaccinates pregnant women who present to antenatal care.* [Line 69]

In response to the reviewer’s query about a seasonal program in Sierra Leone - indeed, a seasonal maternal pneumococcal vaccination programs may be more cost-effective in settings with clinically significant seasonality in invasive pneumococcal disease. The seasonality of pneumococcal disease has been demonstrated in African, Asian, and American settings [16, 17]. A recent study of the seasonality of pneumococcal transmission in Gambia – another Western African nation – found the prevalence of pneumococcal carriage to be higher during the dry than the rainy season (57.6% compared to 47.8% (p<0.001)[18]. The absolute difference in prevalence of pneumococcal between dry and rainy seasons, while statistically significant, is unlikely to determine the impact of a maternal pneumococcal vaccination program in Sierra Leone.

**Comment 6: There appears to be very little uncertainty in the model looking at Table 3, could the authors comment on this? I.e. how good is the inherent uncertainty in the model in capturing the true uncertainty? Again, perhaps creating the plots described above might help answer this point.**

We have thoroughly examined how uncertainty is propagated through the model, and detailed changes in our response to Reviewer 1 Comment 5.

**Comment 7: Tables - Could the DALY estimates be added to a table with uncertainty (maybe 1 or 2)?**

We have included a table with DALY estimates in the Supplementary Material (Table S1). We have included DALY estimates with three levels of discounting (0%, 3%, 5%) to demonstrate the impact of ongoing discounting on the valuation of ongoing health outcomes. We have not included uncertainty in these estimates since this uncertainty can not be analytically calculated. Our model samples from distributions fit to the uncertainty of parameters underlying the estimation of DALYs, e.g., duration of symptomatic infection and severity of lifelong sequalae, for each individual in each simulation.

*(Excerpt from the Supplementary Material – page 3)*

# *S2. DALY estimates*

*Table S1 presents point estimates for DALYs by severity of pneumococcal infection. Our model samples from the distributions underlying these point estimates during each model run.*

***Table S1.*** *DALYs associated with different outcomes of pneumococcal infection.*

| ***Outcome*** | ***DALYs*** |
| --- | --- |
| *Death* | *55.92 (0% discounting)*  *27.11 (3% discounting)*  *18.78 (5% discounting)* |
| *Lifelong meningitis sequalae* | *7.5 (0% discounting)*  *3.6 (3% discounting)*  *2.5 (5% discounting)* |
| *Non-fatal invasive pneumococcal disease* | *0.0036* |
| *Non-fatal non-invasive pneumococcal disease* | *0.0014* |

**Comment 8: Figure 1 - Could point-estimate proportions be added to the lines for Figure 1? This would help give a diagrammatic overview of the cost-effectiveness model.**

We have considered this suggestion, however, our estimates for the severity of pneumococcal infection vary are age specific. Unfortunately, it is not possible to clearly display the distribution of the twenty-six age classes across the health outcomes represented in Figure 1.

**Comment 9: Figure 2 - Add grid lines to the plot. Remove the underscores from the legend. Label the dashed line.**

We have amended our figure to include these requests.

*(Excerpt from the Results)*

***Fig 3. Tornado diagram representing one-way sensitivity analysis.*** *The influence of key model parameters on incremental cost-effectiveness per DALY averted (2020 USD). Dashed line represents Sierra Leone’s GDP per capita [5].*

**Reviewer #3**

**Comment 1: In the abstract: “95% prediction interval” rather than “range”.**

We have included this revision.

*(Excerpt from the Abstract)*

*Our model estimates that a maternal pneumococcal program would cost $606 (2020 USD, 95% prediction interval 437 to 779) from a healthcare perspective and $132 (95% prediction interval -1 to 265) from a societal perspective per DALY averted for one year of vaccine delivery.* *[Line 27]*

**Comment 2: In the abstract: for the results you are mentioning, specify “over one year of vaccine delivery” or “for one year of vaccine delivery” since the benefit of vaccine protection is lifelong and thus not limited to the hypothetical year of implementation.**

We have included this revision.

*(Excerpt from the Abstract)*

*Our model estimates that a maternal pneumococcal program would cost $606 (2020 USD, 95% prediction interval 437 to 779) from a healthcare perspective and $132 (95% prediction interval -1 to 265) from a societal perspective per DALY averted for one year of vaccine delivery.* *[Line 27]*

**Comment 3: In the abstract: “the choice of discounting rates for health outcomes determines” => specify that this is discounting “over individuals’ life” (maybe find a better way to say this). Just to avoid any confusion with “discounting over the period of vaccine delivery”, which is something different and not what you are doing in the paper as I understand it.**

We have included this revision.

*(Excerpt from the Abstract)*

*Sensitivity analysis demonstrates how the choice to discount ongoing health benefits determines whether the maternal pneumococcal vaccine was deemed cost-effective from a healthcare perspective. [Line 34]*

**Comment 4: I would mention the dynamic transmission model earlier in the paper (e.g. in the Introduction or the abstract). Otherwise some readers might discard the paper too fast, thinking it is just using a static model.**

We have included additional text in the abstract and introduction as suggested.

*(Excerpt from the Abstract)*

*In this paper, we use results from a dynamic transmission model to assess the cost-effectiveness of a maternal pneumococcal polysaccharide vaccine from a health sector perspective. [Line 21]*

*(Excerpt from the Introduction)*

*In a previous paper, we constructed a dynamic transmission model which demonstrated that a maternal pneumococcal vaccine could reduce incidence by 73% (range 49-88%) in children <1 month, and 55% (range 36-66%) in children 1-2 months old [7]. [Line 62]*

**Comment 5: Line 72. Remove “, so” ?**

This sentence was rewritten to address revisions suggested by Reviewer 1 and 2.

**Comment 6: Line 87. “There is high uptake of a maternal tetanus vaccine in Sierra Leone, with 97.4% of pregnant women receiving at least one dose during pregnancy [14]. A single dose of PPV is sufficient [16].” => This part sounds a bit strange. Do you mean that you will assume a 97.4% coverage for PPV based on the maternal tetanus vaccine coverage, and that PPV is a single dose vaccine?**

We acknowledge that our language was confusing and have clarified our wording to better reflect our meaning.

*(Excerpt from the Methods)*

*As vaccination with PPV requires a single dose [19], we estimated maternal pneumococcal vaccine coverage from the 2019 Demographic Health Survey estimate that 97.4% of pregnant woman in Sierra Leone receive at least one dose of maternal tetanus vaccine [14]. [Line 104]*

**Comment 7: Line 97. “For children under 1”. Are indirect effects on older age groups negligible? Is this something you found in your previous study?**

Yes, the reviewer is correct. We have added a sentence to the Methods to make this explicit.

*(Excerpt from the Methods)*

*Our transmission model focused on examining the effects of a maternal vaccine on children under one year of age since maternally derived immunity is expected to wane after one year [20]. We did not consider indirect effects of a maternal vaccine on older age groups since previous modelling suggested that a maternal pneumococcal vaccine would not cause widespread or ongoing reductions in transmission in the community [7]. [Line 119]*

**Comment 8: Line 108. First use of “IPD” => define it here.**

We have included this revision.

*(Excerpt from the Methods)*

*We employed the ‘severe lower respiratory infection’ weight (0.133; 95% CI 0.088-0.190) for all non-fatal invasive pneumococcal disease episodes, and ‘moderate lower respiratory infection’ weight (0.051; 95% CI 0.032-0.074) for all non-invasive pneumococcal disease episodes. [Line 134]*

**Reference List**

1.	World Health Organization. WHO guide for standardization of economic evaluations of immunization programmes. Geneva: World Health Organization, 2019 2019. Report No.: Contract No.: WHO/IVB/19.10.

2.	Kobayashi M, Abdul-Karim A, Milucky JL, Zakariah A, Leidner AJ, Asiedu-Bekoe F, et al. Estimating the economic burden of pneumococcal meningitis and pneumonia in northern Ghana in the African meningitis belt post-PCV13 introduction. Vaccine. 2021;39(33):4685-99. Epub 20210701. doi: 10.1016/j.vaccine.2021.06.043. PubMed PMID: 34218962.

3.	The World Bank. World Bank Open Data <https://data.worldbank.org/2022> [cited 2022 22/11/2022]. Available from: <https://data.worldbank.org/>.

4.	World Population Prospects 2019 [Internet]. 2019 [cited 26/11/2021]. Available from: <https://population.un.org/wpp/Download/Standard/Mortality/>.

5.	International Monetary Fund. World Economic Outlook Database: April 2021 Edition 2021 [07/10/2021]. Available from: <https://www.imf.org/en/Publications/WEO/weo-database/2021/April>.

6.	Holmlund E, Nohynek H, Quiambao B, Ollgren J, Kayhty H. Mother-infant vaccination with pneumococcal polysaccharide vaccine: Persistence of maternal antibodies and responses of infants to vaccination. Vaccine. 2011;29(28):4565-75. doi: 10.1016/j.vaccine.2011.04.068. PubMed PMID: WOS:000292471700005.

7.	Bilgin GM, Lokuge K, Glass K. Modelling the impact of maternal pneumococcal vaccination on infant pneumococcal disease in low-income settings. Vaccine. 2022;40(31):4128-34. Epub 20220603. doi: 10.1016/j.vaccine.2022.05.066. PubMed PMID: 35667913.

8.	Touray MML, Hutubessy R, Acharya A. The cost effectiveness of pneumococcal conjugate vaccine in the routine infant immunisation programme of The Gambia. Journal of Pharmaceutical Health Services Research. 2011;2(3):175-84. doi: 10.1111/j.1759-8893.2011.00057.x. PubMed PMID: WOS:000215342600007.

9.	Ayieko P, Griffiths UK, Ndiritu M, Moisi J, Mugoya IK, Kamau T, et al. Assessment of health benefits and cost-effectiveness of 10-valent and 13-valent pneumococcal conjugate vaccination in Kenyan children. PLoS One. 2013;8(6):e67324. Epub 20130624. doi: <https://doi.org/10.1371/journal.pone.0067324>. PubMed PMID: 23826268; PubMed Central PMCID: PMCPMC3691111.

10.	Ojal J, Griffiths U, Hammitt LL, Adetifa I, Akech D, Tabu C, et al. Sustaining pneumococcal vaccination after transitioning from Gavi support: a modelling and cost-effectiveness study in Kenya. Lancet Glob Health. 2019;7(5):e644-e54. Epub 2019/04/20. doi: 10.1016/S2214-109X(18)30562-X. PubMed PMID: 31000132; PubMed Central PMCID: PMCPMC6484775.

11.	Kebede TT, Svensson M, Addissie A, Trollfors B, Andersson R. Cost-effectiveness of childhood pneumococcal vaccination program in Ethiopia: results from a quasi-experimental evaluation. BMC Public Health. 2019;19(1):1078. Epub 20190809. doi: 10.1186/s12889-019-7423-8. PubMed PMID: 31399030; PubMed Central PMCID: PMCPMC6688319.

12.	Fox-Rushby JA, Hanson K. Calculating and presenting disability adjusted life years (DALYs) in cost-effectiveness analysis. Health Policy Plan. 2001;16(3):326-31. Epub 2001/08/31. doi: 10.1093/heapol/16.3.326. PubMed PMID: 11527874.

13.	World Health Organization. WHO-CHOICE estimates of cost for inpatient and outpatient health service delivery. 2021.

14.	Statistics Sierra Leone & ICF. Sierra Leone Demographic Health Survey 2019. Freetown, Sierra Leone, and Rockville, Maryland, USA2020.

15.	Bertram MY, Lauer JA, De Joncheere K, Edejer T, Hutubessy R, Kieny MP, et al. Cost-effectiveness thresholds: pros and cons. Bull World Health Organ. 2016;94(12):925-30. Epub 2016/12/21. doi: 10.2471/blt.15.164418. PubMed PMID: 27994285; PubMed Central PMCID: PMCPMC5153921.

16.	Gessner BD, Mueller JE, Yaro S. African meningitis belt pneumococcal disease epidemiology indicates a need for an effective serotype 1 containing vaccine, including for older children and adults. BMC Infectious Diseases. 2010;10(1):22. doi: 10.1186/1471-2334-10-22.

17.	Numminen E, Chewapreecha C, Turner C, Goldblatt D, Nosten F, Bentley SD, et al. Climate induces seasonality in pneumococcal transmission. Scientific Reports. 2015;5(1):11344. doi: 10.1038/srep11344.

18.	Bojang A, Jafali J, Egere UE, Hill PC, Antonio M, Jeffries D, et al. Seasonality of Pneumococcal Nasopharyngeal Carriage in Rural Gambia Determined within the Context of a Cluster Randomized Pneumococcal Vaccine Trial. PLOS ONE. 2015;10(7):e0129649. doi: 10.1371/journal.pone.0129649.

19.	Merck Sharp & Dohme Corporation. Pneumovax23 Prescribing Information. New Jersey, USA2021.

20.	Zimmermann P, Perrett KP, Messina NL, Donath S, Ritz N, van der Klis FRM, et al. The Effect of Maternal Immunisation During Pregnancy on Infant Vaccine Responses. EClinicalMedicine. 2019;13:21-30. Epub 20190726. doi: 10.1016/j.eclinm.2019.06.010. PubMed PMID: 31517260; PubMed Central PMCID: PMCPMC6733996.
